# Declining ecosystem health and the dilution effect

**DOI:** 10.1038/srep31314

**Published:** 2016-08-08

**Authors:** Hussein Khalil, Frauke Ecke, Magnus Evander, Magnus Magnusson, Birger Hörnfeldt

**Affiliations:** 1 Department of Wildlife, Fish, and Environmental Studies, Swedish University of Agricultural Sciences, Skogmarksgränd, SE-901 83 Umeå, Sweden; 2 Department of Aquatic Sciences and Assessment, Swedish University of Agricultural Sciences, Gerda Nilssons väg 5, SE-756 51 Uppsala Sweden; 3 Department of Clinical Microbiology, Virology, Umeå University, SE-901 85 Umeå, Sweden

## Abstract

The “dilution effect” implies that where species vary in susceptibility to infection by a pathogen, higher diversity often leads to lower infection prevalence in hosts. For directly transmitted pathogens, non-host species may “dilute” infection directly (1) and indirectly (2). Competitors and predators may (1) alter host behavior to reduce pathogen transmission or (2) reduce host density. In a well-studied system, we tested the dilution of the zoonotic Puumala hantavirus (PUUV) in bank voles (*Myodes glareolus*) by two competitors and a predator. Our study was based on long-term PUUV infection data (2003–2013) in northern Sweden. The field vole (*Microtus agrestis*) and the common shrew (*Sorex araneus*) are bank vole competitors and Tengmalm’s owl (*Aegolius funereus*) is a main predator of bank voles. Infection probability in bank voles decreased when common shrew density increased, suggesting that common shrews reduced PUUV transmission. Field voles suppressed bank vole density in meadows and clear-cuts and indirectly diluted PUUV infection. Further, Tengmalm’s owl decline in 1980–2013 may have contributed to higher PUUV infection rates in bank voles in 2003–2013 compared to 1979–1986. Our study provides further evidence for dilution effect and suggests that owls may have an important role in reducing disease risk.

Land use change and habitat destruction contribute to loss of biodiversity and disruption of natural processes^1^. Disturbed ecosystems become “unhealthy”^2^ when hosts and vectors become dominant in depleted communities^3^,^4^. Ecosystem disturbance is thought to particularly affect zoonotic pathogens, i.e. those shared between humans and vertebrate animals, which comprise a majority of emerging infectious diseases of humans^5^. As human activities contributing to “unhealthy” ecosystems continue to accelerate^6^, interest in the role of diversity and community composition in modifying disease risk is growing^7^.

In disease systems where species vary in their susceptibility to infection by a pathogen, higher diversity often results in lower disease risk (reviewed in ref. 8). This is termed “the dilution effect”^3^ and acts on processes at different levels of the disease-cycle. The dilution effect framework in zoonotic systems was developed for the tick-borne Lyme disease system^9^. A key component of the dilution effect is that species-assemblages are nested, where reservoir hosts (those that maintain and transmit the pathogen) persist at low diversity^10^,^11^. Habitat specialist, predators, or species with a slow life history disappear from disturbed areas, while reservoir hosts tend to be habitat generalists, have fast life histories, and tolerate disturbance^12^,^13^,^14^. For example, in Central and South America, agricultural activities result in changes in the composition of rodent assemblages, which become restricted to few species. Those species that persist are often hosts for hantaviruses and their dominance of agricultural and peri-domestic areas increases human risk^13^.

The strength, scale, and generality of the dilution effect have been debated, but most caveats pertain to vector-borne pathogen systems^15^,^16^,^17^. For vector-borne pathogens with multiple hosts, complexities may arise if an increase in vector density associated with high species diversity counteracts the dilution effect^18^. However, community assembly is typically substitutive so that when diversity increases, individuals are replaced rather than added to maintain a constant total density in the community. Since total host density remains constant, vector density is unlikely to increase when diversity increases^19^. For directly-transmitted zoonotic viruses such as hantavirus, transmission rates and disease risk are not confounded by a vector, and the dilution effect depends on changes in host density or behavior^20^.

Puumala hantavirus (PUUV, family *Bunyaviridae*, genus *Hantavirus*) is a single-stranded RNA virus that causes hemorrhagic fever with renal syndrome in humans^21^. The natural and only competent host of PUUV, i.e. capable of furthering the infection cycle through shedding of viral particles upon infection^22^, is the bank vole (*Myodes glareolus*)^23^. It is a very common mammal in Europe^24^, and despite bank vole preference for forest habitats, it can reach high densities in other habitat types^25^ and often prevails at low species diversity. In Fennoscandina, its population and that of other small mammals undergo synchronous 3–4 year cycles^26^,^27^,^28^,^29^. PUUV is directly and horizontally transmitted within bank vole populations and viral particles are shed in the saliva, feces, and urine^30^. Human PUUV infections correlate with bank vole density and infection rates^31^,^32^,^33^,^34^,^35^ and have increased in the past decade both in Northern and Western-Central Europe^35^,^36^.

There are two mechanisms by which non-host species, including predators, may reduce PUUV infection in bank voles (reviewed in ref. 37). (1) The “encounter reduction” pathway occurs if non-host species change the behavior of bank voles, ultimately reducing encounter rate or duration between infected and susceptible individuals^38^. (2) The “susceptible host regulation”^38^ acts through suppression of bank vole density^39^. PUUV prevalence, i.e. proportion of infected bank voles in a population, has often been found to be density-dependent, e.g.^31^,^40^,^41^, so reduction in host density reduces PUUV transmission and prevalence among bank voles.

The potential of other species to dilute PUUV infection in bank voles is under-explored (but see refs 41 and 42). However, there is strong support for the dilution effect in hantavirus-host systems in North and Central America. Through experimental and observational studies, several studies reported lower Hantavirus infection rates in hosts at higher diversity of small mammals, e.g. refs 43, 44, 45, 46, 47. In a heterogeneous landscape where the bank vole and other small mammals fluctuate synchronously^26^,^27^,^28^,^48^, the relationship between different modes of inter-specific interactions and PUUV infection in bank voles is not trivial. To evaluate the validity of the dilution effect (see Fig. 1 in ref. 37), we use long-term data and account for habitat-specific, seasonal, and annual PUUV infection patterns.

In our study area in northern Sweden, small mammals have been monitored since 1971^26^. The bank vole is the most common species and can be found in most habitats^25^,^49^. The grey-sided vole *Myodes rufocanus* is the main competitor of the bank vole in coniferous forests^50^ and has declined and locally disappeared in the early 2000’s^51^,^52^. The field vole (*Microtus agrestis*) has also declined since the 1970’s (Figure S1a)^25^, yet persists in the landscape mainly in open areas dominated by grasses in the field layer, e.g. meadows and clear-cuts^52^. It is competitively superior to the bank vole and may exclude it from clear-cuts and young forests^25^. Hence, the field vole could cause a “dilution effect” due to its ability to affect both bank vole behavior and survival^53^. The common shrew (*Sorex araneus*) is a competitor and nest predator of bank voles^54^. This solitary small-sized insectivore can be found in most habitat types^55^. Recent studies have shown that the presence of common shrews influences the behavior and home range of lactating female bank voles^54^,^56^. Thus, the common shrew may dilute PUUV infection in bank voles through influencing bank vole behavior. While the grey-sided and field voles declined^52^, bank voles increased during the last decade (Figure S1), suggesting that drivers causing the decline in other vole species have not equally affected bank vole populations (Fig. 1).

Tengmalm’s owl (*Aegolius funereus*) is a predator specializing on small mammals, and field and bank voles constitute approximately 85% of its diet^57^. Nest box occupancy of breeding Tengmalm’s owls in the study area has declined since its monitoring began in 1980 and continues to fluctuate at low levels^58^. In theory, predators of hosts may reduce disease risk both by selectively taking infected host individuals^59^ and by regulating host density^60^. Empirical work on predation and dilution of infection is scarce, but Tengmalm’s owls probably suppress bank vole density^61^,^62^.

Here, we investigate the dilution effect in a well-studied system of a directly-transmitted zoonotic pathogen (PUUV) in boreal Sweden^28^,^31^,^49^,^52^,^63^. We hypothesize that both field voles and common shrews will dilute PUUV infection in bank voles through changing their movement patterns and reducing contact rates, i.e. cause a dilution effect via “encounter reduction”. Moreover, we expect field voles, but not common shrews, to indirectly reduce PUUV infection by suppressing bank vole densities, i.e. via “susceptible host regulation”. Effects of field voles on bank vole density and PUUV prevalence should be strongest in core field vole habitat. To test our hypotheses, we used long-term trapping data over a large area, while incorporating habitat at a local patch scale. Although areas of owl nest box monitoring and small mammal trapping only partially overlap, we discuss how long-term decline of Tengmalm’s owls may have affected PUUV infection and host density in 2003–2013.

## Results

**Figure 1:**
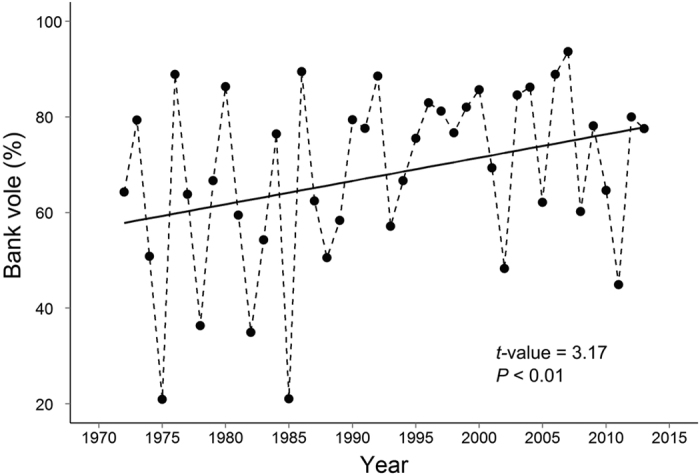
Percentage of number of bank voles out of all trapped small mammals in spring in 1971–2013.

In 2003–2013, trapped small mammals in our analyses consisted of 4169 bank voles (84% of all trapped specimens), 545 field voles (11%) and 271 common shrews (5%). In total 942 bank voles were infected, and overall PUUV prevalence was 22.5%. Overall PUUV prevalence in spring (47%) was higher than in fall (17%). In 1971–2013, the percentage of bank voles relative to total number of small mammals increased in both spring (Fig. 1, *t*-value = 3.17, *p* < 0.01, df_residual_ = 41) and fall (*t*-value = 2.03, *p* = 0.04, df_residual_ = 41).

### Encounter reduction

**Figure 2:**
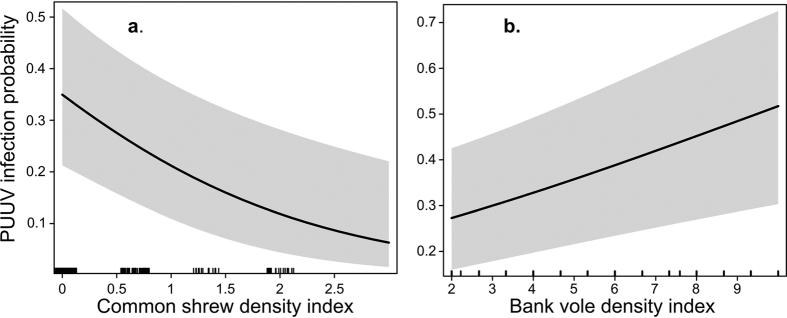
The model-predicted probability of a bank vole being Puumala virus-infected in spring. Relative to (**a**) common shrew density index and (**b**) bank vole density index. The grey-shaded area represents the 95% confidence interval of coefficient estimates. Vertical black marks on the x-axis show how predictor values are distributed across predictor range, denser marks indicate a concentration of predictor values.

**Figure 3:**
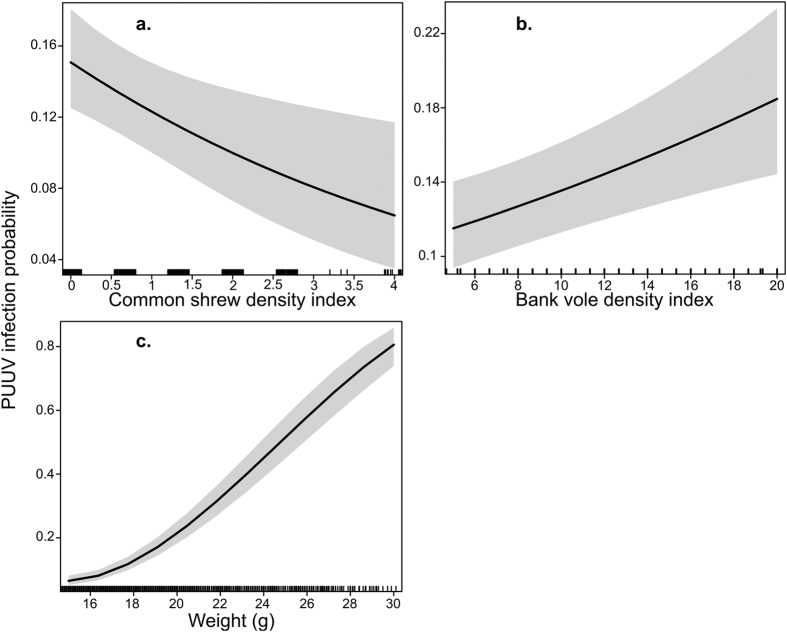
The model-predicted probability of a bank vole being infected in fall. Relative to (**a**) common shrew density index, (**b**) bank vole density index and (**c**) weight (g). The grey-shaded area represents the 95% confidence interval of coefficient estimates. Vertical black marks on the x-axis show how predictor values are distributed across predictor range, denser marks indicate a concentration of predictor values.

In both spring and fall, the best model (Table S1, models 1 and 2) predicting the probability of a bank vole being infected included common shrew density index and bank vole density index as predictors (Table 1, Figs 2 and 3). Infection probability increased with bank vole density index. However, infection probability decreased as common shrew density index increased. In fall, the best model suggested that infection probability also increased with bank vole weight (Table 1, Fig. 3). Neither habitat nor field vole density index influenced infection probability in either season, despite field voles showing a higher overall density index compared with common shrews (Figure S1).

### Susceptible host regulation

**Figure 4:**
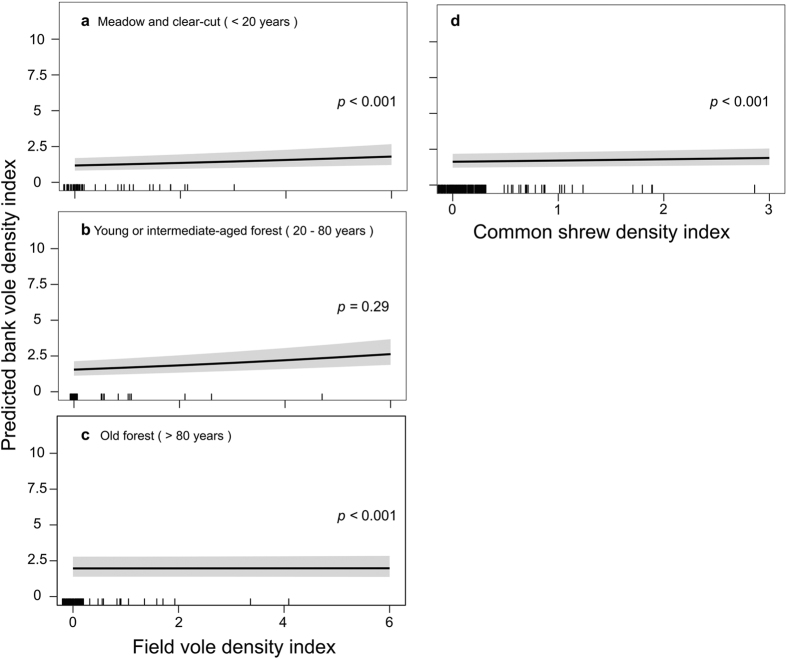
Model-predicted bank vole density index in spring. Relative to (**a–c**) field vole density index in different habitat succession stages, and relative to (**d**) common shrew density index. The grey-shaded area represents the 95% confidence interval of coefficient estimates. Vertical black marks on the x-axis (rug plots) show how predictor values are distributed across predictor range, denser marks indicate a concentration of predictor values.

**Figure 5:**
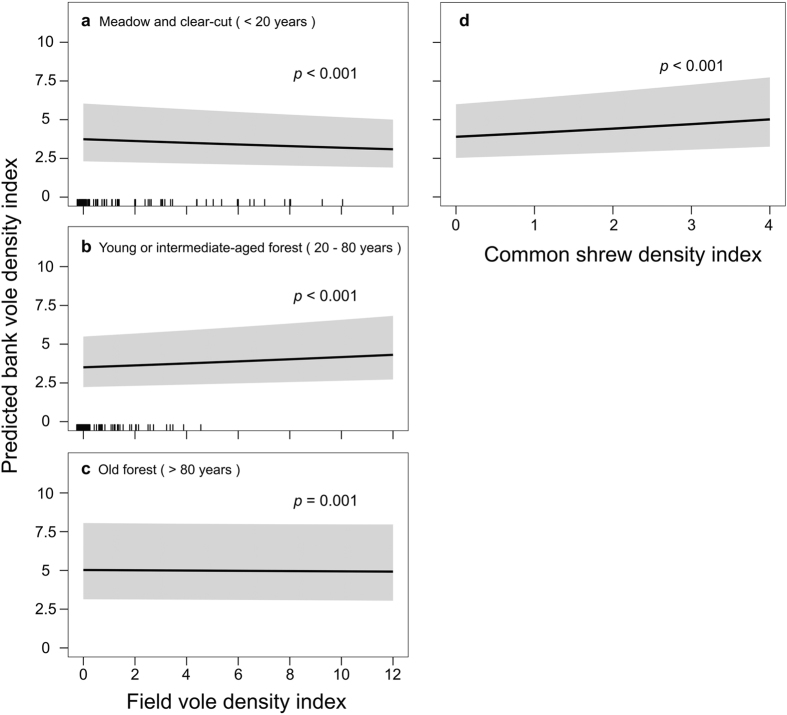
Model-predicted bank vole density index in fall. Relative to (**a–c**) field vole density index in different habitat succession stages, and relative to (**d**) common shrew density index. The grey-shaded area represents the 95% confidence interval of coefficient estimates. Vertical black marks on the x-axis (rug plots) show how predictor values are distributed across predictor range, denser marks indicate a concentration of predictor values.

Factors predicting bank vole density index were similar in spring and fall models, but the direction of the relationships differed (Table S1, models 3 and 4). In both spring and fall, bank vole density index increased with common shrew density index, irrespective of habitat (Table 1). Current bank vole density index was negatively related to its previous density index (Year_t-1_) (Table 1), and was higher in older forest compared to young and intermediate-aged forests and meadows and clear-cuts. There was an interaction between field vole density index and habitat in both seasons, but the direction of the relationship differed between spring and fall. In spring, bank vole density index increased as field vole density index increased in meadows and clear-cuts and intermediate-aged forests (Fig. 4a,b). In fall, we found the opposite scenario, and bank vole density index decreased when field vole density increased, but only in core field vole habitat, i.e. meadows and clear-cuts (Fig. 5a).

**Figure 6:**
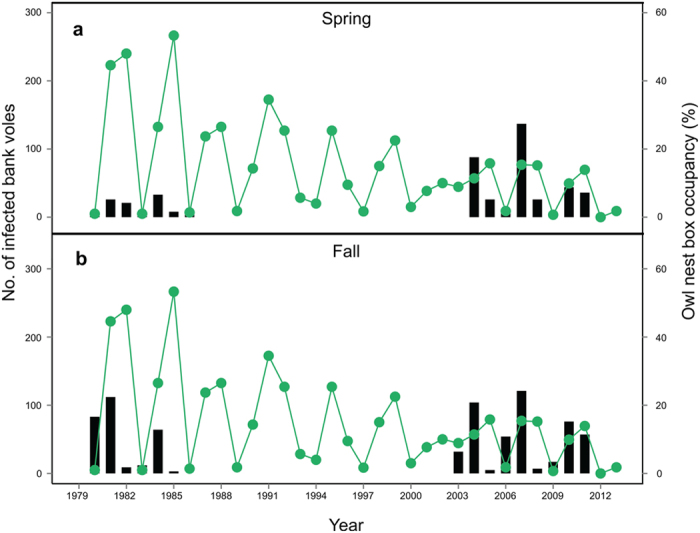
The number of infected bank voles (bars and left-hand y-axis) in (**a**) spring and (**b**) fall in two time periods: 1979–1986 and 2003–2013 and Tengmalm’s owl nest box occupancy (%) (line and right-hand y-axis) in spring in 1980–2013.

Owls nest box occupancy (%) decreased in 1980–2013 (Fig. 6, t-value = −5.4, *p* < 0.001, df_residual_ = 32). Concurrently, the number of infected voles *per* cycle was higher in the 2003–2013 time-frame compared to that in 1979–1986. This difference was most evident in spring (Fig. 6a). In 1979–1986, there were 413 infected bank voles (206.5 *per* cycle), whereas there were 942 infected in 2003–2013 (314 *per* cycle). Also, mean prevalence in spring was 7% higher in 2003–2013 than in 1979–1986.

## Discussion

As far as we know, this study is the first to investigate PUUV dilution by non-host small mammals through species-specific hypotheses. The probability of infection in a bank vole decreased with increasing common shrew density index (Figs 2 and 3). In addition, the field vole affected PUUV prevalence indirectly by suppressing bank vole density index in fall in meadows and clear-cuts. The decrease in nest box occupancy of Tengmalm’s owl during the past three decades was concurrent with an increase in overall density of infected voles and prevalence in spring in the 2003–2013 time-frame compared to that in 1979–1986. Our study thus found evidence for the dilution effect by two non-host species, and suggested that Tengmalm’s owls are important in reducing PUUV infection in bank voles.

Our results are part of a growing *corpus* of evidence for the dilution of hantavirus infection in a range of new and old world hantavirus-host systems. For example, in an experimental study in Panama, both infection prevalence and host density increased when small mammal diversity was reduced^43^. In the United States, Dizney and Dearing^44^ found that hosts of Sin Nombre hantavirus in more diverse sites spent less time engaged in behaviors related to pathogen transmission and were less likely to be infected. Similar results were found in Argentina in an observational study, as host individuals infected with Andes hantavirus were more likely to be found near human dwellings where small mammal diversity was low^64^. In Europe, Voutilainen *et al.*^41^ found evidence for the dilution of PUUV infection in bank voles through pooling densities of non-host small mammals. Here, by studying the potential of common shrews and field voles to influence PUUV infection in bank voles independently, we were able to infer mechanisms and conditions that promote dilution of PUUV.

The common shrew is found in a wide range of habitats^55^. It is smaller and competitively inferior than the bank vole^48^,^65^. They are unlikely to regulate bank vole densities and we found that the two species densities were positively related (Figs 4b and 5d). Correlated changes in density indices were expected due to the synchronous population fluctuations of small mammals regionally^48^. Nevertheless, dilution through “encounter reduction” reduces infection in host populations irrespective of host density^47^. In an experimental study, the presence of common shrews changed bank vole behavior, resulting in lactating females visiting fewer supplementary feeding stations^56^. Common shrews are opportunistic predators and may prey on vole nestlings, and the two species share above ground runways and tunnels^54^. As a response to risk, bank voles may avoid common shrews and increase time spent protecting nestlings. Ultimately, we expect that a reduction in movement of infected voles limited the spatial scale of PUUV shedding and number of encounters with susceptible voles. In North America, the short-tailed shrew (*Blarina brevicauda*) restricts spatial use of the meadow vole (*Microtus pennsylvanicus*)^66^ and may prey on it^67^. Dilution of PUUV through encounter reduction was also reported from Western Europe. In Belgium, PUUV prevalence in bank voles was lower when non-host wood mouse (*Apodemus sylvaticus*) density increased relative to bank voles^42^. Further, direct evidence for encounter reduction came from the Sin Nombre hantavirus system. Based on an experimental setup, Clay *et al.*^47^ reported that contact rates among hosts declined when non-host diversity increased.

Alternatively, competition can indirectly reduce infection prevalence by reducing host density. PUUV prevalence increased with bank vole density index in spring and fall (Figs 3 and 4), likely due to accelerated density-dependent transmission^68^. Nevertheless, infection prevalence was higher in spring than in fall despite fall density indices being higher. This is probably due to the influx of uninfected newborn voles into the population, which masks the increase in density-dependent transmission^69^. Field voles suppressed bank vole density in meadows and clear-cuts in fall (Fig. 5a), when bank vole density is often highest. In the reproductive season, field vole populations reach peak densities after bank voles^48^. Competition between the two species was most likely space-driven after reproduction^70^ and we detected interference competition by field voles only in our fall data (Table 1). Also, winter survival in field voles has declined, leading to lower spring densities^28^,^52^ and thus reduced spring competition between the two species. Bank voles may reach high densities in meadows^71^, but interference competition from field voles limits bank vole density^53^, and thereby PUUV transmission. Only in core field vole habitats - where field voles are more abundant than in other habitat types^70^ - bank vole density index declined as that of the field vole increased (Fig. 5a). Because field voles also alter bank vole behavior^53^, we expected field voles to also directly reduce PUUV infection in bank voles in meadows and clear-cuts. But we found no evidence for dilution through “encounter reduction” (Table S1, models 1 and 2). We speculate that space-driven interference competition occurred for a limited time-period after reproduction, outside of which bank vole behavior, encounter rates, and PUUV transmission were not sufficiently altered to be reflected in PUUV infection rates.

In meadows and clear-cuts and intermediate-aged forests in spring and in intermediate and old-aged forests in fall, bank voles and field vole density indices were positively related. Bank vole and common shrew density indices were correlated irrespective of habitat type or season. Fairly synchronous fluctuations in density are typical of cyclic small mammals in northern Fennoscandia^27^,^48^, suggesting common external drivers such as predators and food availability that synchronize fluctuations of small mammal species^29^, ultimately overwhelming competitive interactions. The negative relationship between field vole and bank vole density indices in meadows and clear-cuts in fall despite the synchronizing forces acting on the different species strengthens the evidence for “susceptible host regulation” hypothesis.

Tengmalm’s owls nest box occupancy declined in 1980–2013. Out of the three vole species that constituted >90% of Tengmalm’s owl diet, i.e. bank vole, field vole, and grey-sided vole^57^, only bank vole density index increased in the 2000’s (Figure S1). PUUV prevalence and infected bank vole density index in spring were higher in 2003–2013 compared to 1979–1986 (Fig. 6). We hypothesize that low field vole and grey-sided vole density indices contributed to Tengmalm’s owl persistent low numbers^72^. The negative relationship between PUUV prevalence (and number of infected bank voles) and owl decline suggests that Tengmalm’s owls may limit infection in bank vole populations. However, this relationship merits further investigation at spatially appropriate scales.

The study area is heavily managed by forestry^52^,^73^ with a species-poor small mammal community^27^. The drastic decline of the grey-sided vole^51^, driven by habitat loss^52^, probably released the bank vole from competition in forest habitats and allowed the latter to expand its niche (*sensu*^50^, Fig. 1). The decline in field voles, to which climate change was suggested to contribute^28^,^74^ may further increase utilization of meadows and clear-cuts by bank voles (Fig. 5). Competitive release of bank voles in new habitats may be associated with higher density and PUUV prevalence, especially in places where virus survival outside the host or transmission may be enhanced due to micro-habitat properties^41^. Identification of micro-habitat factors, e.g. resource distribution and structural and physical properties would facilitate predicting PUUV dynamics in habitats where the bank vole replaces its competitors.

Our results are based on long-term time series collected systematically, over a large area with plots 2.5 km apart. This enabled us to test the dilution effect at the mechanistically important local (plot) scale, while accounting for habitat differences. It is at the plot level where changes in bank vole density and behavior are expected to affect PUUV infection within populations. Also, the simple system with directly transmitted pathogen and few non-host small mammal species enabled us to include density indices of non-host species rather than species richness. Nevertheless, our inferences of dilution mechanisms were based on observational data. Experimental testing in large enclosures is needed to establish a direct link between behavioral and density changes in bank voles (e.g. refs 53, 75 and 76) to changes in transmission rates. For example, experimental work on the dilution effect is ongoing in the United States on Sin Nombre virus system (reviewed by ref. 77).

We highlighted the role of non-host species in directly and indirectly reducing PUUV infection prevalence in bank voles. We found evidence for the dilution effect by a competitor (field vole) that conditionally regulated bank vole density indices thereby indirectly reducing PUUV infection, and a nest predator (common shrew) that directly influenced bank vole infection probability. The long-term decline in Tengmalm’s owls coincided with a general increase in density indices and infection prevalence in bank voles in 2003–2013, and thus higher number of infected voles (Fig. 6). The increase in infected bank voles, including our study period 2003–2013, points to an increasing human risk in Northern Sweden. Our results provide evidence for the importance of functional diversity in a given community in reducing pathogen infection in hosts. Landscape and climate changes may increase risk of hantavirus infections in humans, especially if a generalist (here the bank vole) dominates when its competitors and predators decline.

## Materials and Methods

### Small mammal and habitat data

Small mammal data in 1971–2013 was available through the ongoing Swedish National Environmental Monitoring Program for small rodents, initiated in 1971 around Umeå in northern Sweden (64° N, 20° E)^27^. The area belongs to the middle boreal zone^78^. Within a 100 ×100 km area, trapping of small mammals takes place twice a year in 58 systematically placed 1-ha plots of at least 2.5 km inter-distance. Spring trapping is in late May whereas fall trapping is in late September. Each 1-ha plot is trapped for three nights along a 90 m line with 10 trapping stations. Each station has five snap traps placed within a 1 m radius circle. The total trapping effort was 150 trap nights *per* plot (see refs 27 and 28 for further details). For each species, a density index was calculated as number of individuals *per* 100 trap nights.

We characterized sampling plots in 2012–2013 according to habitat type and three forest succession stages: meadows and clear-cuts <20 years (n = 12), young and intermediate-aged forest 20–80 years (n = 24), and old forest >80 years (n = 14). Two sampling plots were on meadows dominated by grasses in the field layer; for small mammals a habitat type often functionally similar to clear-cuts^79^. The dominant forest age class along the trapping line was used in the analyses and forest age was estimated by increment coring at breast height combined with visual observations.

This study, including small mammal and owl monitoring, was approved by the Animal Ethics Committee in Umeå (Dnr A 11–14, A 12–14 and A 13–14), and all applicable institutional and national guidelines for the use of animals were followed.

### Owl data

Data on Tengmalm’s owls breeding was collected since 1980 from nest boxes placed in trees at approximately 1 km interval in an area partially overlapping with the small mammal monitoring area^57^. We used nest box occupancy data in 1980–2013. The number of nest boxes checked *per* year varied and ranged between 275 and 500^58^,^80^. Breeding attempts were confirmed through systematic visits in spring. Tengmalm’s owl reproduction is largely dependent on vole density^57^ and is reflected in annual variation in box occupancy by breeding owls. Nest box occupancy was calculated as the percentage of boxes occupied.

### Hantavirus infection data

In this study we focused on the 2003–2013 infection data, published for the first time, while we used available infection data in 1979–1986 (n = 2064 bank voles^31^,^81^) for comparison.

We analyzed lung samples from bank voles by enzyme-linked immunosorbent assay (ELISA) to detect anti-PUUV IgG antibodies and identify sero-positive individuals^31^,^82^. Sero-positivity points to an ongoing infection in bank voles since shedding of PUUV is life-long^83^. Thus, we use the term infected rather than sero-positive throughout this paper. Bank voles weighing <14.4 g may carry maternal antibodies^41^,^84^ and were excluded (n = 866) from further analyses since their sero-positivity may not reflect genuine infection. In subsequent analyses, PUUV infection data from 4169 bank voles in 2003–2013 was used.

### Statistical analyses

#### Bank vole dominance

To confirm that bank voles have increased in proportion relative to other small mammals, we calculated the percentage of bank voles relative to total number of small mammals (% bank voles) in spring and fall. The time series of percentage of bank voles showed temporal autocorrelation in both seasons. We hence fitted a generalized least square model with a temporal autocorrelation structure (maximum lag = 3 in spring and 2 in fall) to % bank voles over time in 1971–2013.

### Encounter reduction

We tested whether PUUV infection probability in bank voles in spring and fall (2003–2013) at local plot level was affected by common shrew and field vole density indices. Also, several studies found hantavirus prevalence to increase with host density (e.g. refs 31, 40, 85 and 86, which is common in horizontally-transmitted pathogens^68^. So we included bank vole density index as a predictor of infection probability at plot level in the analysis. We also included local habitat (meadows and clear-cuts, young and intermediate-aged forest, and old forest) since habitat influences PUUV dynamics (e.g. refs 41 and 81). Probability of PUUV infection often increases with weight, a surrogate of bank vole age (e.g. refs 40 and 87), so weight (g) was also used as a predictor.

We fitted a generalized linear mixed effects model with a binomial error distribution to predict the probability of a bank vole being infected. Models for spring and fall were fitted independently as there was little overlap between the two seasons in the ranges of predictors (density index and weight). The response was binary: infected versus uninfected. Candidate fixed effects were bank vole density index, common shrew density index, field vole density index, bank vole weight (g), and habitat. Plot identity and year were included as random effects. We did not have data on bank vole sex, hence we could not test for sex differences in infection probability. Often, males are more likely to be infected with hantaviruses than females^40^,^88^. However, we do not expect sex differences in infection probability to influence our results in relation to the dilution effect.

#### Susceptible host regulation

We found that PUUV infection probability increased with bank vole density index (results). Hence, we investigated whether the common shrew and the field vole indirectly reduced PUUV infection by regulating bank vole density index at plot level. Also, we included bank vole density index in the previous year as a predictor to account for delayed-density dependence^27^,^28^. We included the interaction between habitat on one hand and field vole density indicex on the other to account for differences in interaction outcomes at different forest succession stages (meadows and clear-cuts, young and intermediate-aged forests, and old forests). Hence, we fitted a generalized linear mixed effects model with a poisson distribution error with bank vole density index as response variable. Candidate predictors were field vole density index, common shrew density index, previous bank vole density index (Year_t-1_), habitat, and the interaction between habitat and field density indices. Plot identity and year were included as random effects.

Tengmalm’s owl nest boxes did not entirely overlap with bank vole trapping areas. We thus did not formally test the relationship between nest box occupancy (%) and PUUV infection in bank voles. However, we discuss how the temporal patterns in owl occupancy (%) in 1980–2013 were related to changes in bank vole PUUV infection between 1979–1986 and 2003–2013. The infection data from 1979–1986 covered two vole cycles whereas 2003–2013 infection data covered three cycles. We fitted a generalized least square model with temporal autocorrelation (maximum lag = 3) to the time series of owl nest box occupancy to determine if it declined. We related the temporal change in nest box occupancy to PUUV prevalence and number of infected voles *per* cycle between the two different time periods (1979–1986 *versus* 2003–2013).

All analyses were performed in R using the “nlme”^89^ and “lme4”^90^ packages in R^91^. All models were checked for violations of assumptions and correlation among explanatory variables. Model residuals were checked for patterns to investigate model fit. Selection of best models was based on AICc criteria. If two or more models had a ∆AICc < 2, only significant predictors were included. Significance was assumed below a probability *p* < 0.05.

## Additional Information

**How to cite this article**: Khalil, H. *et al.* Declining ecosystem health and the dilution effect. *Sci. Rep.* **6**, 31314; doi: 10.1038/srep31314 (2016).

## Supplementary Material

Supplementary Information

## Figures and Tables

**Table 1:** Models predicting Puumala virus-infection probability in bank voles and bank vole density index.

**Infection probability *binomial***							
	Fall				Spring		
	***OR***	***CI***	***P***		***OR***	***CI***	***P***
Fixed Parts							
Bank vole density	1.04	1.02–1.06	**<0.001**	Bank vole density	1.14	1.05–1.24	**0.001**
Common shrew density	0.79	0.67–0.94	**0.007**	Common shrew density	0.50	0.32–0.78	**0.002**
Weight (g)	1.31	1.27–1.35	**<0.001**				
Random Parts							
N_plots_	54			N_plots_	53		
N_Year_	11			N_Year_	10		
ICC_plots_	0.017			ICC_plots_	0.060		
ICC_Year_	0.015			ICC_Year_	0.203		
Observations	3330			Observations	839		
Bank vole density *poisson*							
Fixed Parts							
Intercept	3.79	2.35–6.14	**<0.001**	Intercept	1.31	0.91–1.88	**<0.001**
Old forest	1.34	0.97–1.87	0.08	Old forest	1.67	1.19–2.33	**0.003**
Young forest	0.94	0.70–1.26	0.69	Young forest	1.31	0.97–1.78	0.08
Bank vole density (t-1)	0.99	0.99–0.99	**<0.001**	Bank vole density (t-1)	0.95	0.95–0.96	**<0.001**
Field vole density	0.98	0.98–0.99	**<0.001**	Field vole density	1.07	1.04–1.11	**<0.001**
Common shrew density	1.07	1.06–1.07	**<0.001**	Common shrew density	1.05	1.03–1.07	**<0.001**
Field vole density × Young forest	1.03	1.02–1.04	**<0.001**	Field vole density × Young forest	1.02	0.98–1.05	0.29
Field vole density × Old forest	1.01	1.01–1.02	**0.001**	Field vole density × Old forest	0.93	0.90–0.96	**<0.001**
Random Parts							
N_plots_	50			N_plots_	48		
N_Year_	11			N_Year_	11		
ICC_plots_	0.05			ICC_plots_	0.05		
ICC_Year_	0.13			ICC_Year_	0.06		
Observations	430			Observations	247		

The reference (intercept) is bank vole density index in meadows and clear-cuts. OR = odds ratio, CI = confidence interval, ICC = intra-class correlation coefficient.
