# Supplementary material for: Declining ecosystem health and the dilution effect
Source: Sci Rep. 2016 Aug 8;6:31314. doi: 10.1038/srep31314 (PMC4976314; doi:10.1038/srep31314)
Supplement: Supplementary Information [file srep31314-s1.pdf]

Declining ecosystem health and the dilution effect

Hussein Khalil, Frauke Ecke, Magnus Evander, Magnus Magnusson, Birger Hörnfeldt

Supplementary information

Figure S1

Small mammal density index in spring (no. trapped individuals *per* 100 trap nights) in 1971-2013. (**a**) Field vole, (**b**) common shrew, (**c**) bank vole. The shaded area represents time periods where Puumala hantavirus infection data is available.

Table S1

Selection of best models that predict infection probability in bank voles and bank vole density index in spring and fall. Selection was based on adjusted Akaike information criterion (AICc) and grey-shaded rows delineate the selected model. If two or more models had a ∆AICc < 2, only significant predictors were included. Only the top four models are shown. Df = degrees of freedom.

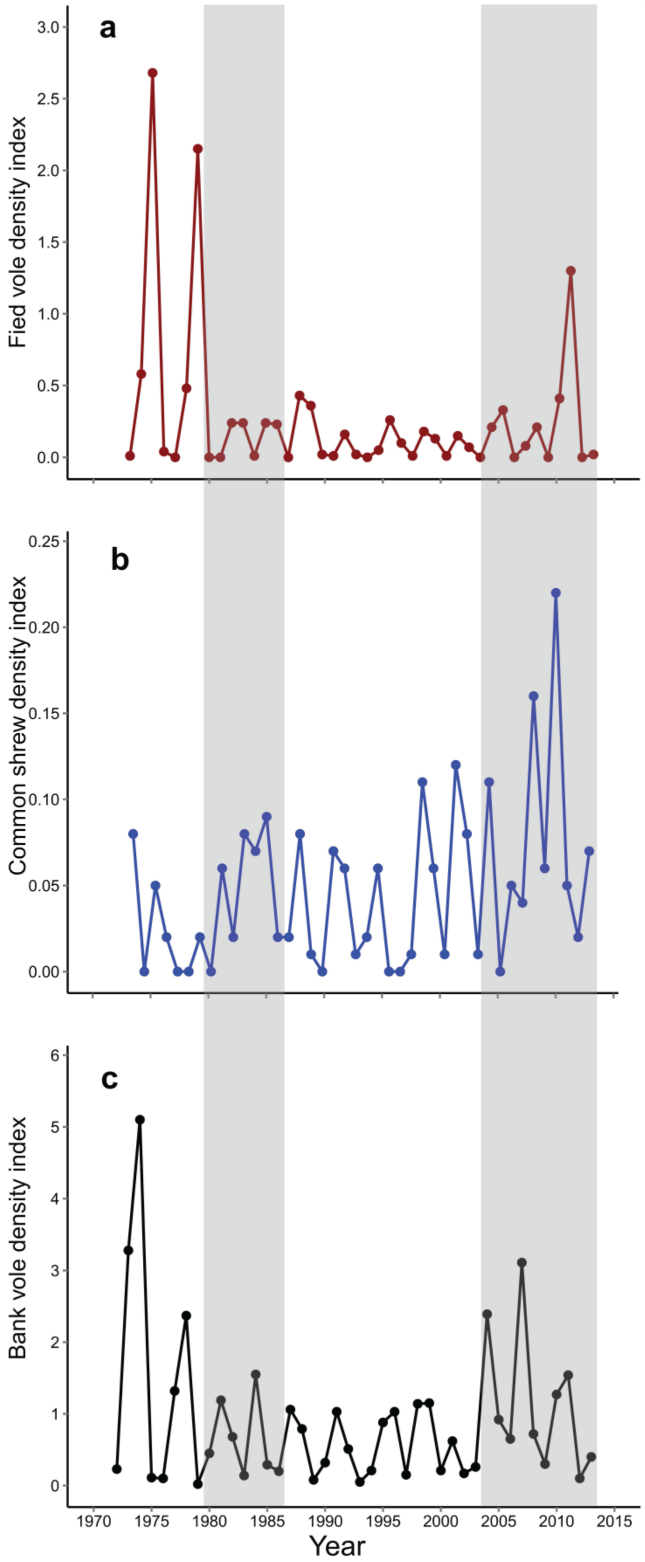
a
Fied vole density index
3.0
2.5
2.0
1.5
1.0
0.5
0.0
b
Common shrew density index
0.25
0.20
0.15
0.10
0.05
0.00
c
Bank vole density index
6
5
4
3
2
1
0
1970
1975
1980
1985
1990
1995
2000
2005
2010
2015
Year

Model No.	Bank vole infection probability In spring<br>Bank vole density index	Forest age class	Field vole density index	Common shrew density index	Weight (g)	Df	AICc	∆AICc	Model weight
**1**	✓			✓		5	956.4	na	0.33
	✓			✓	✓	6	957.2	0.87	0.22
	✓		✓	✓		8	958.4	1.97	0.12
	✓	✓		✓		8	959.3	2.85	0.10
	**Bank vole infection probability In fall**								
	✓	✓		✓	✓	8	2361.7		0.45
**2**	✓			✓	✓	6	2363.1	1.48	0.22
	✓		✓	✓	✓	9	2625.6	1.90	0.17
	✓	✓	✓	✓	✓	7	2626.9	3.45	0.10

	Bank vole density index in spring<br>Previous bank vole density index	Forest age class	Field vole density index	Common shrew density index	Forest age class × Field vole density index	Df	AICc	∆AICc	Model weight
**3**	✓	✓	✓	✓	✓	10	19512	na	1
	✓	✓	✓		✓	9	19530	18.27	0
	✓	✓	✓	✓		8	19568	56.3	0
	✓		✓	✓		6	19571	59.5	0
	**Bank vole density index in fall**								
	✓	✓	✓	✓	✓	10	60128	na	1
**4**	✓	✓	✓	✓		9	60182	53.7	0
	✓		✓	✓		7	60184	55.7	0
	✓	✓		✓		8	60214	86	0
